# The effect of surgical management of endometrioma on the IVF/ICSI outcomes when compared with no treatment? A systematic review and meta-analysis

**DOI:** 10.1007/s00404-017-4640-1

**Published:** 2018-01-17

**Authors:** M. Nickkho-Amiry, R. Savant, K. Majumder, E. Edi-O’sagie, M. Akhtar

**Affiliations:** 1 University Hospital of South Manchester, Southmoor Rd, Wythenshawe, Manchester M23 9LT UK; 2 Central Manchester Foundation Trust, Manchester, UK

**Keywords:** Endometrioma, Surgery, ART, Pregnancy outcome

## Abstract

**Objective:**

To assess the impact of surgical management of endometrioma on the outcome of assisted reproduction treatment (ART).

**Design:**

A systematic review and meta-analysis.

**Setting:**

Department of reproductive medicine at teaching university hospital, UK.

**Patients:**

Subfertile women with endometrioma undergoing ART.

**Interventions:**

Surgical removal of endometrioma or expectant management.

**Main outcome measures:**

Clinical pregnancy rate, pregnancy rate, live birth rate, number of oocytes retrieved and number of embryos available and ovarian response to gonadotrophins.

**Results:**

An extensive search of electronic databases for articles published from inception to September 2016 yielded 11 eligible studies for meta-analysis. Meta-analysis was conducted comparing surgery versus no treatment of endometrioma. There were no significant differences in pregnancy rate per cycle, clinical pregnancy rate and live birth rate between women who underwent surgery for endometrioma and those who did not.

**Conclusion:**

Current evidence suggests that women with endometriosis-related infertility have similar cycle outcomes to other patients going through ART. It is pertinent for clinicians to assess the risks of surgical intervention on ovarian reserve prior to initiating therapy.

## Introduction

Endometriosis is a chronic-debilitating disease that affects 5–10% of fertile women [1]. It is characterised by the presence of endometrial-like tissue (glands and stroma) outside the uterus, which induces a chronic inflammatory reaction, scar tissue, and adhesions that may distort a woman’s pelvic anatomy [2]. Around 25–50% of women with infertility may be affected by endometriosis, and 30–50% of women with endometriosis have infertility [3].

Women with endometriosis often require assisted reproduction technology (ART) and the severity of endometriosis has been linked to ART outcome [4]. However, further research is necessary to understand this association. Multiple hypotheses have been suggested to explain the low fecundity observed with endometriosis. Most commonly, the association has been attributed to altered folliculogenesis resulting in reduced quality oocytes [5], mechanical interference with oocyte pickup and transportation [6], exposure to a hostile environment of macrophages, cytokines and vasoactive substances in the peritoneal fluid [7, 8] and anatomical dysfunction of the fallopian tube and ovary [9].

An endometrioma is the formation of a cyst within the ovary with ectopic endometrial tissue lining [10, 11]. An endometrioma is one of the most common manifestations of endometriosis. Endometriomas are found in 17–44% of patients with endometriosis [12]. The pathogenesis of an endometrioma is complex and different compared to that of other benign ovarian cysts. A majority of endometriomas are thought to be pseudocysts as described by Hughesdon rather than intra-ovarian cysts [10, 11].

Endometriomas are often associated with deep endometriosis and often do not respond well to medical therapy. Medical therapy may relieve the symptoms and improve pain or reduce the size of the cyst but does not improve infertility [13]. Therefore, the focus has been on surgical treatment in an attempt to improve fertility.

There has been much speculation as to the exact mechanism by which endometriomas cause infertility. Researchers have suggested that there is a decrease in ovarian reserve and follicular density in women with endometriomas possibly due to an increase in oxidative stress [14]. However, surgical resection of these cysts has been shown to further decrease ovarian reserve [13]. This highlights that there is much debate regarding the treatment of endometriomas, and uncertainty with regards to infertility, particularly in women who are undergoing assisted reproductive technology (ART).

The aim of this paper is to elucidate the effect of surgical management of ovarian endometriomas on fertility outcomes after ART.

## Materials and methods

### Search strategy

Related studies were identified after extensive search of PUBMED, Medline, EMBASE and Cochrane database from inception to September 2016. The following keywords and synonyms were used: ‘endometrioma’, ‘cystectomy’, ‘IVF’, ICSI’, ‘pregnancy’. The language of publication was restricted to English. The European Society of Human Reproduction and Embryology guidelines were also reviewed. International standard randomised controlled trial number registry was checked for any trials registered with them. The reference lists of all publications and reviews were hand-searched to identify missing relevant publications. Two authors (RS and MA) independently conducted the search, and reviewed titles, abstracts and full manuscripts. Each article was independently assessed for inclusion and exclusion criteria. The review was registered with PROSPERO: International prospective register of systematic reviews. The ID number is CRD42015023914.

### Study selection

The studies that were included in the meta-analysis met the following criteria: (1) an original paper; (2) a study of ovarian endometrioma; (3) a clinical study (including randomised controlled trials, case–control, prospective and retrospective cohort studies) that assessed the association of ovarian response, oocyte quality, embryo quality and IVF outcome with ovarian endometrioma.

All controlled retrospective or prospective studies that studied the effect of surgery on endometrioma or aspiration of endometrioma on IVF/ICSI outcome and ovarian response to gonadotrophins and those with a defined comparison group were included in the review.

The major exclusion criteria were literature reviews, non-original articles; non-ovarian endometrioma; duplication of a previous publication; and women who did not receive intervention on the endometrioma and women who had received medical or surgical treatment of their ovarian endometrioma before IVF cycles.

**Fig. 1:**
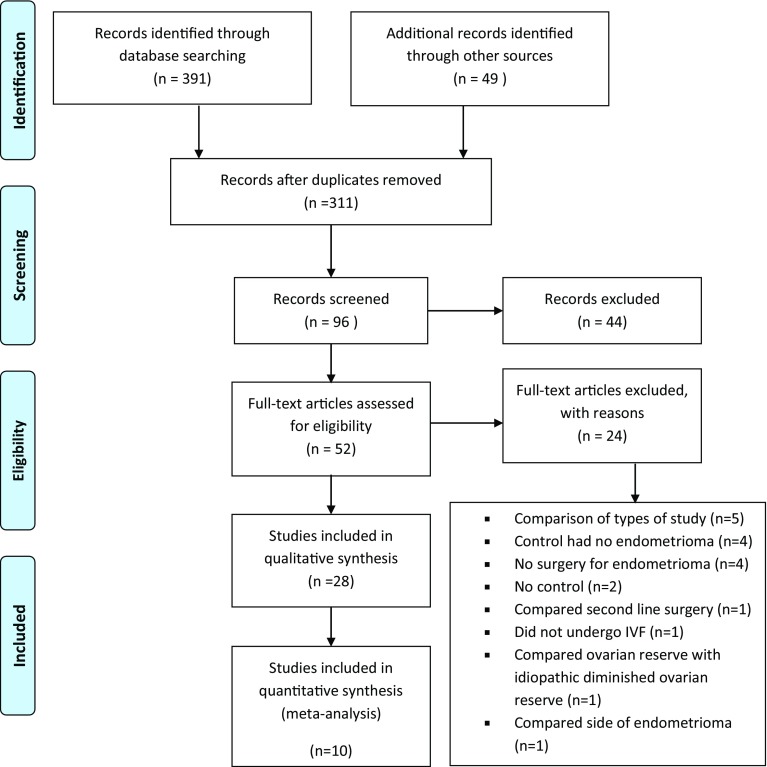
Prisma flow diagram

The Preferred Reporting Items for Systematic Reviews and Meta-Analyses (PRISMA) checklist was used while writing this review (Fig. 1).

### Outcome measures

The primary outcomes were live birth rate per cycle, clinical pregnancy rate per cycle (defined as visualisation of fetal heart activity on transvaginal ultrasound at ≥ 6 week) and pregnancy rate (positive pregnancy test after ART).

Secondary measures included the ovarian response to gonadotropin stimulation by the total number of gonadotrophin ampoules required for ovarian stimulation, the peak E2 levels on the day of the hCG administration and the total number of oocytes retrieved with and number of embryos available for transfer.

### Statistical analysis

Data analyses were carried out using RevMan, version 5.3 (Cochrane, Collaboration, Oxford, UK). Heterogeneity was evaluated graphically using forest plots and statistically using the *I*2 statistic to quantify heterogeneity across studies. An *I*2 > 50% was considered to represent substantial heterogeneity between studies. A random-effect model was used for meta-analysis in cases of high heterogeneity, and a fixed- effect model was used in cases of low heterogeneity. Dichotomous outcome data were reported as odds ratios with 95% confidence intervals (CI). Continuous data were synthesized using weighted means with 95% CI for variables including number of gonadotrophin ampoules required for ovarian stimulation, the peak E2 level and the number of oocytes.

## Results

The search strategy yielded 721 articles, 91 of these were relevant to our review. 48 of these studies were found to be potentially eligible.

### Included studies

The characteristics of the 28 studies included in the systematic review and classified according to their controls are presented in Table 1. The majority were retrospective case–control studies. Ten studies were included for quantitative synthesis that compared surgical treatment versus no treatment (meta-analysis, Fig. 1). There were two prospective cohort [15, 16] and three retrospective cohort studies [17–19]. Only one randomised control trial was available for study. Randomisation for aspiration of endometrioma was done in one study [15]. One prospective case–control study with randomisation for gonadotrophins was noted [20].

**Table 1:** Characteristics of all studies included in the systematic review

Study (reference)	Design	Intervention	Study group	Control group	Type of surgery	Cyst size (cm)	Laterality	Dt	Outcomes
*Control group: non-treated endometrioma*									
Tinkanen and Kujansuu [30]	Retrospective case–control	IVF-ET long protocol	Surgical treatment of endometrioma	Non-treated endometrioma	Endometrioma stripping	1.5–7	Either	1–7 years	NOR, no. of embryos, FR, IR, PR, LBR
Suganuma et al. [21]	Retrospective case–control	IVF-ET long protocol	Surgical treatment of endometrioma	Aspirated endometrioma Non-treated endometrioma	Endometrioma stripping (laparoscopy or laparotomy)	ND	ND	31 ± 27 months	NOR, FR, PR
Garcia-Velasco et al. [29]	Retrospective case–control	IVF-ET long protocol	Surgical treatment of endometrioma	Non-treated endometrioma	Laparoscopic ovarian cystectomy	> 3	Unilateral	12 months	NOR, no. of embryos, FR, IR, CPR, MR, units, E2 peak
Pabuccu et al. [24]	Prospective cohort Randomised for aspiration	IVF/ICSI long protocol	Surgical treatment of endometrioma	Non-treated endometrioma Aspirated endometrioma Tubal factor infertility	Endometrioma stripping (laparoscopy or laparotomy)	ND	Either	≤ 4 years	Mature follicles, FR, IR, CPR, MR, ampoules, E2 peak
Wong et al. [19]	Retrospective cohort	IVF/ICSI long protocol	Surgical treatment of endometrioma	Non-treated endometrioma Non-treated peritoneal endometriosis	Laparoscopic cystectomy	ND	ND	3–48 months	Mature follicles, FR, IR, PR, CPR, MR, ampoules, E2 peak
Demirol et al. [31]	RCT	ICSI long protocol	Surgical removal of endometrioma	No treatment	Endometrioma stripping (laparoscopy)	3–6	Unilateral	3 months	NOR, FR, IR, CPR, E2 peak
Pabuccu et al. [20]	Prospective case control (Randomised for GnRh agonist/antagonist)	IVF/ICSI long protocol	Surgical removal of endometrioma	No treatment Non-treated peritoneal endometriosis	Endometrioma (laparoscopy or laparotomy)		Either	< 4 years	Ampoules, NOR, FR, no. of embryos, CPR
Bongioanni et al. [27]	Retrospective case–control	IVF/ICSI long protocol	Surgical treatment of endometrioma	No Treatment Tubal factor infertility	Endometrioma stripping (laparoscopy)	< 6	Either	< 5 years	FR, NOR, PR, LBR
Lee et al. [28]	Retrospective case–control	IVF/ICSI long protocol	Surgical treatment of endometrioma	No treatment Aspiration with ethanol sclerotherapy	Laparoscopic cystectomy	4–7	ND	20 months	NOR,CPR, LBR
Dong et al. [18]	Retrospective cohort	IVF/ICSI long protocol	Surgical treatment of endometrioma	No treatment	Laparoscopic cystectomy	ND	ND		NOR, no. of embryos, CPR, LBR
*Control: aspirated endometrioma*									
Suganuma et al. [21]	Retrospective case–control	IVF-ET long protocol	Surgical treatment of endometrioma	Aspirated endometrioma Non-treated endometrioma	Endometrioma stripping (laparoscopy or laparotomy)	ND	ND	31 ± 27 months	NOR, FR, PR
Takuma et al. [22]	Retrospective case–control	IVF-ET long protocol	Surgical treatment of endometrioma	Aspirated endometrioma	Endometrioma stripping (laparoscopy or laparotomy	ND	ND	31 ± 27 months	NOR, FR, PR
Pabuccu et al. [15]	Prospective cohort randomised for aspiration	IVF/ICSI long protocol	Surgical treatment of endometrioma	Non-treated endometrioma Aspirated endometrioma Tubal factor infertility	Endometrioma stripping (laparoscopy or laparotomy)	ND	Either	≤ 4 years	Mature follicles, FR, IR, CPR, MR, ampoules, E2 peak
Lee et al. [28]	Retrospective case–control	IVF/ICSI long protocol	Surgical treatment of endometrioma	No treatment Aspiration with ethanol sclerotherapy	Laparoscopic cystectomy	4–7	ND	20 months	NOR,CPR, LBR
Aflatoonian et al. [62]	RCT	IVF/ICSI long protocol	Ethanol sclerotherapy	No treatment	N/A	3.5–5.5	ND	3	NOR, no. of embryos, ampoules, FR, CPR
*Control group: tubal factor infertility*									
Marconi et al. [17]	Retrospective cohort	IVF-ET long protocol	Surgical treatment of endometrioma	Tubal factor infertility	Endometrioma stripping (laparoscopy	4.8 ± 2.3	Either	Either 12 ± 7 months	Mature follicles, NOR, CPR, ampoules, E2 peak
Wu et al. [69]	Retrospective case–control	IVF-ET long protocol	Surgical treatment of endometrioma	Contralateral normal ovary	Laparoscopic cystectomy	3.9 ± 1.5	Unilateral	2.4 ± 1.7 months	Mature follicles, NOR, no. of embryos, IR, CPR, ampoules, E2 peak
Wyns and Donnez [25]	Retrospective case–control	IVF-ET long protocol	Surgical treatment of endometrioma	Tubal factor infertility Laparoscopically treated peritoneal endometriosis Idiopathic infertility Contralateral normal ovary	Laparoscopic cyst wall laser vaporization	ND	ND	ND	Mature follicles, number of embryos, FR, IR, CPR, ampoules, E2 peak
Pabuccu et al. [15]	Prospective cohort	IVF/ICSI long protocol	Surgical treatment of endometrioma	Non-treated endometrioma Aspirated endometrioma Tubal factor infertility	Endometrioma stripping (laparoscopy or laparotomy)	ND	Either	≤ 4 years	Mature follicles, FR, IR, CPR, MR, ampoules, E2 peak
Loo et al. [66]	Retrospective case–control	IVF-ET long protocol	Surgical treatment of endometrioma	Tubal factor infertility	Laparoscopic cystectomy	> 3	ND	6 months	NOR, no. of embryos, FR, IR, CPR, units, E2 peak
Esinler et al. [63]	Retrospective case–control	ICSI long protocol	Surgical treatment of endometrioma	Tubal factor infertility	Laparoscopic cystectomy	> 3	Either	ND	Mature follicles, IR, CPR, MR, LBR, units, E2 peak
Matalliotakis et al. [67]	Retrospective case–control	IVF/ICSI long protocol	Surgical treatment of endometrioma	Tubal factor infertility	Laparoscopic cystectomy	ND	ND	ND	Mature follicles, NOR, no. of embryos, IR, FR, PR, CPR, MR, LBR, ampoules, E2 peak
Bongioanni et al. [27]	Retrospective case–control	IVF/ICSI long protocol	Surgical treatment of endometrioma	No treatment tubal factor infertility	Endometrioma stripping (laparoscopy)	< 6	Either	< 5 years	FR, NOR, PR, LBR
Kahyaoglu et al. [64]	Retrospective case–control	IVF long protocol	Surgical treatment of endometrioma	Tubal factor infertility	Laparoscopic cystectomy	>3 cm	ND	ND	Ampoules, no. of embryos, FR, CPR,
*Control: laparoscopically treated peritoneal endometriosis*									
Wyns and Donnez [25]	Retrospective case–control	IVF-ET long protocol	Surgical treatment of endometrioma	Tubal factor infertility Laparoscopically treated peritoneal endometriosis Idiopathic infertility Contralateral normal ovary	Laparoscopic cyst wall laser vaporization	ND	ND	ND	Mature follicles, number of embryos, FR, IR, CPR, ampoules, E2 peak
Duru et al. [41]	Retrospective case–control	IVF/ICSI	Surgical treatment of endometrioma	Laparoscopically treated peritoneal endometriosis Contralateral normal ovary	Endometrioma stripping Laparoscopy Laparotomy	ND	Unilateral	≥ 1 year	Mature follicles, CPR
*Control: non-treated peritoneal endometriosis*									
Wong et al. [19]	Retrospective cohort	IVF/ICSI long protocol	Surgical treatment of endometrioma	Non-treated endometrioma Non-treated peritoneal endometriosis	Laparoscopic cystectomy	ND	ND	3–48 months	Mature follicles, FR, IR, PR, CPR, MR, ampoules, E2 peak
Wyns and Donnez [25]	Retrospective case–control	IVF-ET long protocol	Surgical treatment of endometrioma	Tubal factor infertility Laparoscopically treated peritoneal endometriosis Idiopathic infertility Contralateral normal ovary	Laparoscopic cyst wall laser vaporization	ND	ND	ND	Mature follicles, number of embryos, FR, IR, CPR, ampoules, E2 peak
*Control: idiopathic infertility*									
Wyns and Donnez [25]	Retrospective case–control	IVF-ET long protocol	Surgical treatment of endometrioma	Tubal factor infertility Laparoscopically treated peritoneal endometriosis Idiopathic infertility Contralateral normal ovary	Laparoscopic cyst wall laser vaporization	ND	ND	ND	Mature follicles, number of embryos, FR, IR, CPR, ampoules, E2 peak
Wyns and Donnez [25]	Retrospective case–control	IVF-ET long protocol	Surgical treatment of endometrioma	Tubal factor infertility Laparoscopically treated peritoneal endometriosis Idiopathic infertility Contralateral normal ovary	Laparoscopic cyst wall laser vaporization	ND	ND	ND	Mature follicles, number of embryos, FR, IR, CPR, ampoules, E2 peak
*Control group: non-endometriotic benign ovarian cyst.*									
Nargund et al. [68]	Retrospective case–control	IVF-ET long protocol	Surgical treatment of endometrioma	Ovarian cystectomy for simple and dermoid cyst	Cystectomy	ND	Unilateral	ND	Mature follicles, NOR
Nargund et al. [68]	Retrospective case–control	IVF-ET long protocol	Surgical treatment of endometrioma	Ovarian cystectomy for simple and dermoid cyst	Cystectomy	ND	Unilateral	ND	Mature follicles, NOR
*Control group: normal non-operated contralateral ovary*									
Loh et al. [65]	Retrospective case–control	IVF-ET long protocol	Surgical treatment of endometrioma	Contralateral normal ovary	Laparoscopic cystectomy	4.23 ± 2	Either	ND	Mature follicles
Ho et al. [23]	Retrospective case–control	IVF-ET long protocol	Surgical treatment of endometrioma	Contralateral normal ovary	Endometrioma stripping (laparoscopy or laparotomy)	ND	Unilateral	31 ± 27 months	Mature follicles, ampoules, E2 peak
Somigliana et al. [54]	Retrospective case–control	IVF/ICSI long protocol	Surgical treatment of endometrioma	Contralateral normal ovary	Laparoscopic cystectomy	3.9 ± 1.5	Unilateral	2.4 ± 1.7 months	Mature follicles, NOR, no. of embryos, IR, CPR, ampoules, E2 peak
Wyns and Donnez [25]	Retrospective case–control	IVF-ET long protocol	Surgical treatment of endometrioma	Tubal factor infertility Laparoscopically treated peritoneal endometriosis Idiopathic infertility Contralateral normal ovary	Laparoscopic cyst wall laser vaporization	ND	ND	ND	Mature follicles, number of embryos, FR, IR, CPR, ampoules, E2 peak
Ragni et al. [16]	Prospective cohort	IVF/ICSI long protocol	Surgical treatment of endometrioma	Contralateral normal ovary	Endometrioma stripping (laparoscopy	4.0 ± 2.4	Unilateral	2.4 ± 2 years	Mature follicles, OR, FR, IR, CPR, ampoules, E2 peak
Duru et al. [41]	Retrospective case–control	IVF/ICSI long protocol	Surgical treatment of endometrioma	Laparoscopically treated peritoneal endometriosis Contralateral normal ovary	Endometrioma stripping Laparoscopy Laparotomy	ND	Unilateral	≥ 1 years	Mature follicles, CPR
Somigliana et al. [26]	Retrospective case control	IVF/CSI long protocol	Surgical treatment of endometrioma	Normal ovaries	Endometrioma stripping Laparoscopy Laparotomy	4 ± 1.6	Bilateral	3.9 ± 3.4 years	NOR, no. of embryos, CPR
Tang et al. [53]	Retrospective case control	IVF long protocol	Surgical treatment of endometrioma	Contralateral normal ovary	Endometrioma stripping Laparoscopy	< 4 and > 4	Unilateral	ND	NOR, CPR

*Dt* interval between surgery and IVF/ICSI, *ND* not documented, *NOR* number of oocytes retrieved, *FR* fertilisation rate, *IR* implantation rate, *PR* pregnancy rate, *CPR* clinical pregnancy rate, *LBR* live birth rate, *Ampoules* ampoules/unit of gonadotrophins used for ovarian stimulation

Laparoscopic excision of endometriomas by either ovarian cystectomy or stripping of the cyst wall was performed in the majority. Seven studies also involved laparotomies for endometrioma surgery [15, 18–23].

### Ovarian stimulation was with the long protocol in the majority of the cases

The size of the endometriomas, the duration from surgery to IVF and the laterality of the cyst are documented in Table 1. The control group varied and this has been classified in Table 1. Seven studied used multiple control groups [15, 18, 19, 22, 24–26].

There was no significant difference between the study and the control group with regards to the patient characteristics and the other confounding factors.

We included a total of eleven studies in our meta-analysis. Ten studies compared surgical treatment for endometrioma with untreated endometrioma and four studies compared surgical treatment of endometrioma with aspiration of endometrioma. Among these, there were six retrospective case–control [19, 20, 26–29], two retrospective cohort [17, 25] and one prospective case–control studies [18]. One randomised control trial [30] and one prospective cohort study with randomisation for aspiration of endometrioma [15] were studied.

**Fig. 2:**
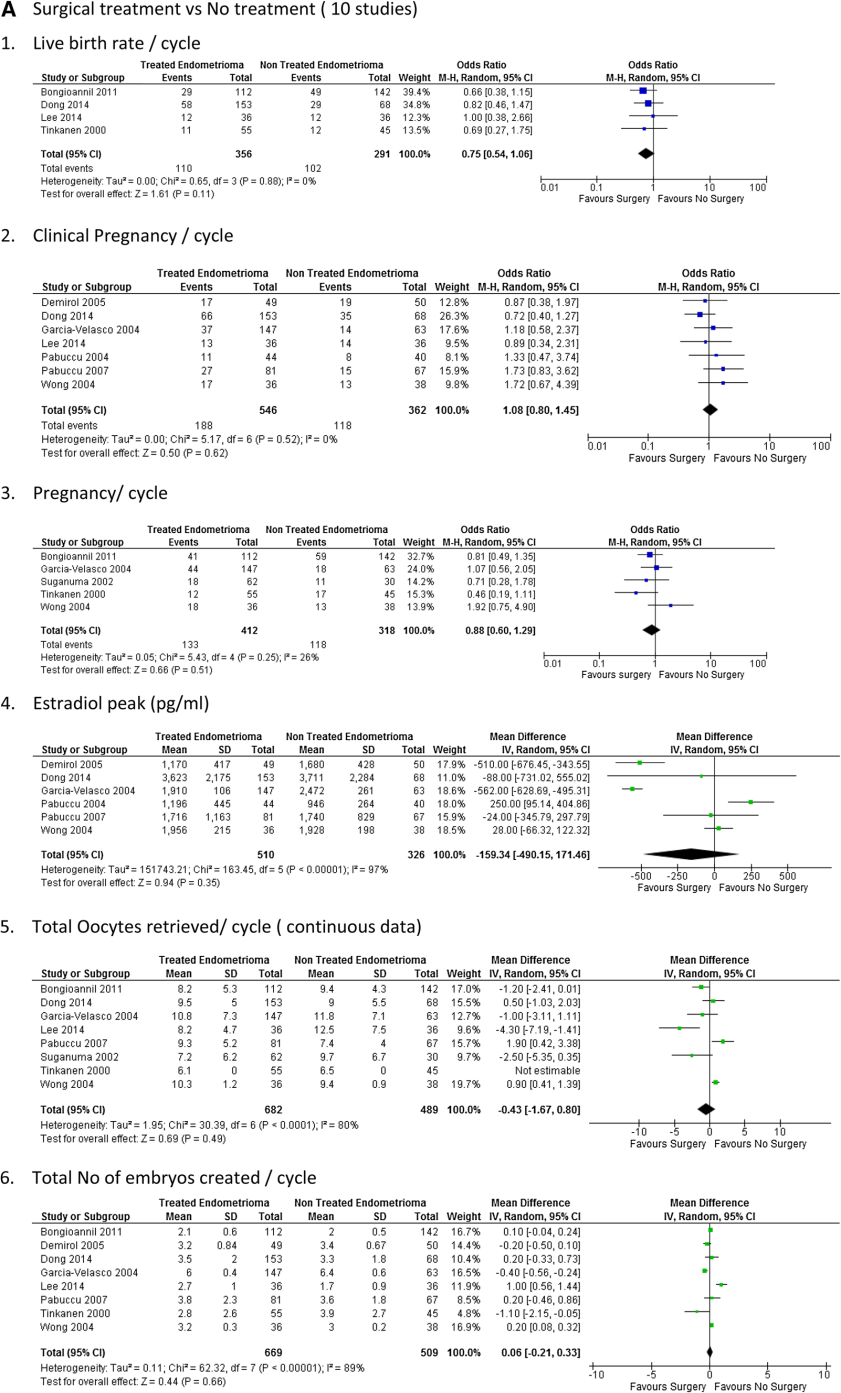

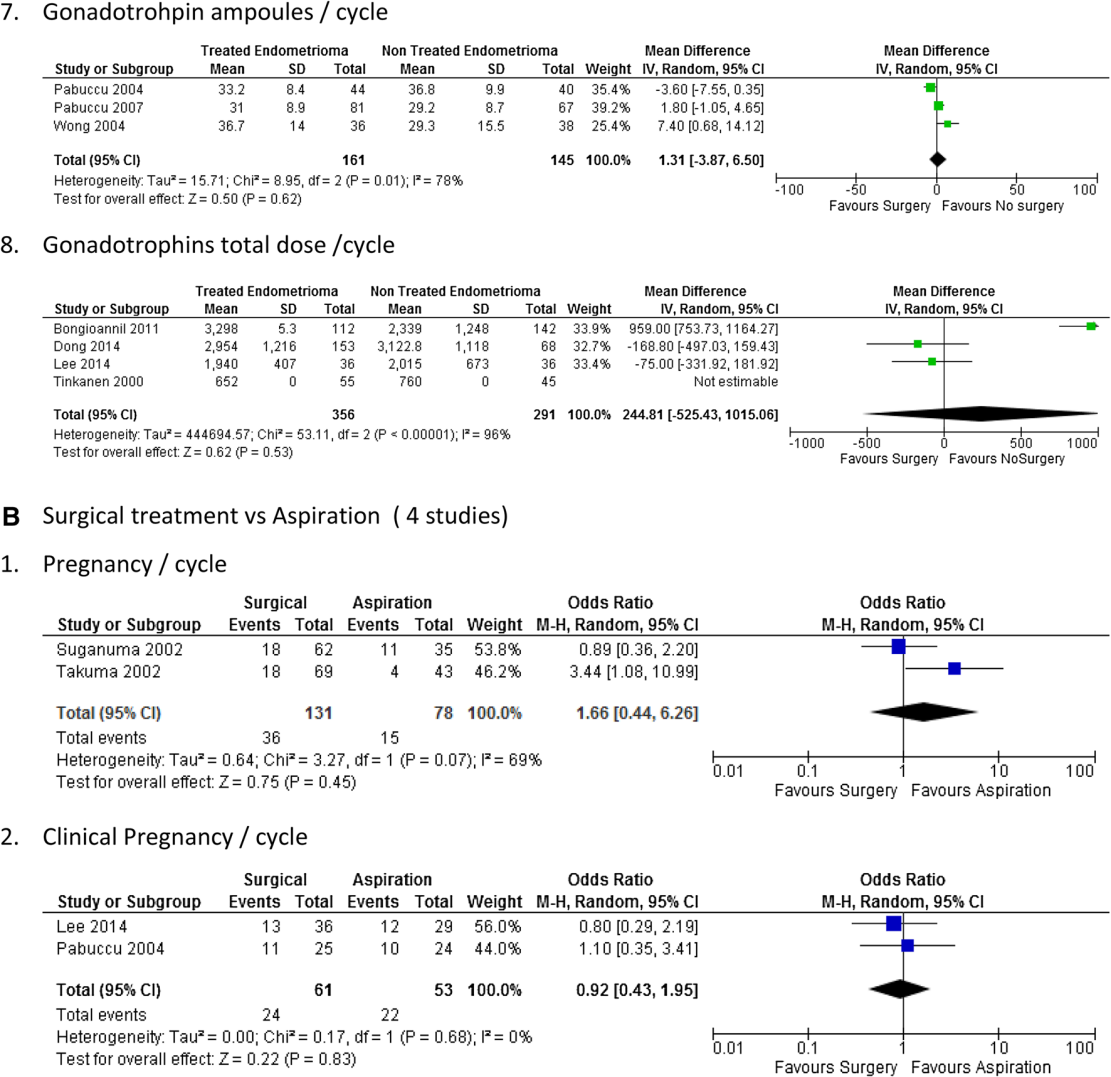
Forest plots examining outcome measures. **a** Surgical treatment versus no treatment (10 studies). 1. Live birth rate/cycle. 2. Clinical pregnancy/cycle. 3. Pregnancy/cycle. 4. Estradiol peak (pg/ml). 5. Total oocytes retrieved/cycle (continuous data). 6. Total no. of embryos created/cycle. 7. Gonadotrophins ampoules/cycle. 8. Gonadotrophins total dose/cycle. **b** Surgical treatment versus aspiration (4 studies). 1. Pregnancy/cycle. 2. Clinical pregnancy/cycle

The forest plots of the meta-analysis comparing surgical treatment with no treatment and surgical treatment with aspiration of endometrioma are presented in Table 2 and Fig. 2.

**Table 2:** Clinical outcomes and parameters of ovarian response assessed in the studies included in the systematic review

Study	Cycles (*n*)		Oocytes retrieved (*n*)		Mature follicles (*n*)		E2 peak (pg/ml)		Implantation rate (%)		Fertilisation rate (%)		Pregnancy rate (%)		Clinical pregnancy rate (%)	
	S	C	S	C	S	C	S	C	S	C	S	C	S	C	S	C
Tinkanen and Kujansuu [30]	55	45	6.1	6.5	ND		ND		13	20	48	58	22	38	ND	
Suganuma et al. [21]	62	30	7.2 ± 6.2	9.7 ± 6.7	ND		ND		ND		56.8	56.5	29	36.6	ND	
	Aspiration of endometrioma ± alcohol fixation 35		6.6 ± 5.5		ND		ND		ND		67.4		31.4		ND	
Takuma [22]	69	43	ND		ND		ND		ND		ND		26	9	ND	
Pabuccu et al. [15]	44	40	ND		5.3 ± 1.2	5.2 ± 1.1	1196 ± 445	946. ± 64	18	12	72 ± 13	68 ± 16	ND		25	20
	Aspirated endometrioma 41		ND		5.9 ± 1.1		1632 ± 670		13		72 ± 10		ND		24	
Garcia-Velasco et al. [29]	147	63	10.8 ± 7	11.8 ± 0.9	ND		191 ± 06	247 ± 61	12.8	14.1	76.5	69.9	30.2	28.8	25.4	22.7
Wong et al. [19]	36	38	10.3 ± 1.2	9.4 ± 0.9	ND		195 ± 15	192 ± 98	20	18	85	88	50	38	47	34
Demirol et al. [31]	49	50	ND		ND		117 ± 17.14	16,80 ± 28.69	16.5	18.5	86.2	88.3	ND		34.4	38.2
Pabuccu et al. [20]	81	67	9.3 ± 5.2	7.4 ± 4	3.1 ± 0.9	4.2 ± 1.5	1716.7 ± 1163.5	1740.3 ± 829.5	19.3	13.6	67.5 ± 21.7	74.6 ± 19.6			33	22.3
Bongioanni et al. [27]	112	142	8.2 ± 5.3	9.4 ± 4.3	ND		ND		24.6	24.2	73.4	67.7	36.6	41.5	ND	
Lee et al. [28]	36	36	8.2 ± 4.7	12. ± .5	3.3 ± 3.0	3.8 ± 2.0	ND		ND		ND		ND		36.1	38.8
	Aspiration with ethanol sclerotherapy 29		12.4 ± 7.6		3.1 ± 1.4										41.3	
Dong et al. [18]	153	68	9.5 ± 5.0	9.0 ± 5.5	8.8 ± 4.2	9.0 ± 4.9	3623.5 ± 2175.3	3711 ± 2284.3	28.3	34.4	58.8	60.7			43.1	51.5

*S* study group, underwent surgical removal or aspiration of endometrioma, *C* control group, *ND* not documented

#### Main outcomes

Surgical treatment compared to no treatment.

There were ten studies that compared surgical treatment to no treatment included in this review. Meta-analysis of these results is as follows:

#### Primary outcome

Live birth rate.

Surgery for endometrioma showed to favour live birth rate per cycle, but this was not statistically significant [4 studies, OR 0.75 (95% CI 0.54, 1.06)] (Fig. 2a1).

#### Clinical pregnancy rate

There were no significant differences in clinical pregnancy rate between women who underwent surgery for endometrioma and those who did not per cycle 1.08 [7 studies, OR 1.08 (95% CI 0.80–1.45)] (Fig. 2a2).

#### Pregnancy rate

There were no significant differences in pregnancy rate per cycle between women who underwent surgery for endometrioma and those who did not [5 studies, OR 0.88 (95% CI 0.60, 1.29)] (Fig. 2a3).

#### Secondary outcomes

##### Number of oocytes retrieved

There was also no statistical difference in the number of oocytes retrieved [mean difference—0.43 (95% CI − 1.67, 0.80)] and the total number of embryos created per cycle [mean difference 0.06 95% CI − 0.21 to 0.33)] in the group that underwent surgery for endometrioma compared to the control with no surgery (Fig. 2a5, a6).

##### Gonadotropin usage

There was no difference in between gonadotrophin ampoules used per cycle [mean difference 1.31 (95% CI (− 3.87, 6.50)] and the total gonadotrophin dose per cycle [mean difference 244.81 (95% CI − 525.43 to 1015.06)] between the two groups (Fig. 2a6, a7).

##### Estradiol peak during ART

There was no difference in the estradiol peak in the two groups. (mean difference − 159.349 (95% CI − 490.15, 171.46)] (Fig. 2a4).

##### Surgical treatment compared to aspiration

There were four studies included in the meta-analysis comparing surgical treatment with aspiration [15, 19, 20, 27].

There was no difference between the pregnancy rate per cycle [OR 1.66 (95% CI 0.44, 6.26)] and clinical pregnancy rate per cycle [OR 0.92 (95% CI − 1.43, 1.95)] between those women who underwent surgery for endometrioma and those who had aspiration of endometrioma (Fig. 2a1, b2). There were no live births reported.

#### Excluded studies

19 studies from the potentially eligible studies were excluded (see Table 3). Four studies were excluded as the control did not have endometrioma and two of the studies did not have a control. Four studies did not have surgery for endometriomas, five compared types of surgery and one compared second line surgery for endometriomas.

**Table 3:** Reason for exclusion of study

Reason for exclusion	References
Control had no endometrioma	[26], [31–34]
No control	[35, 36]
No surgery for endometrioma	[37–40]
Types of surgery	[41–45]
Second-line surgery	[46]
Side of endometrioma	[47]
Did not undergo IVF	[48]
Compared ovarian reserve with idiopathic diminished ovarian reserve	[49]

One studies compared the effect of the side of endometriomas, the subjects did not undergo IVF in another and one studied reduced ovarian reserve in comparison to idiopathic diminished ovarian reserve.

## Discussion

The aim of this systematic review and meta-analysis was to assess the impact of surgical management of endometrioma, on the outcome of assisted reproduction. Our main finding is that there was no significant difference in pregnancy rate per cycle, clinical pregnancy rate and live birth rate between women who underwent surgery for endometrioma and those who did not. Interestingly, there was a slight improvement in live birth rate but only four studies published live birth. The limitation of these data is that most of the studies on surgical management are retrospective in nature and very few publishing data on live birth rate. There is also the added limitation of variations in surgical techniques (i.e., ablation versus resection), completeness of removal of the disease, and differences in ART laboratories.

There is much controversy regarding the surgical management of endometrioma on assisted reproduction outcome. Studies have suggested that the pathophysiologic process in endometrioma formation may be different to other manifestations of endometriosis [11, 14].

Pre-cycle surgical management of endometrioma has been suggested to be beneficial in specific circumstances and include [1] inability to access follicles at oocyte retrieval, [2] concern that oocytes may be exposed to endometrioma fluid, which may damage oocytes, and [3] the presumption that endometrioma resection would improve IVF outcome. These will be addressed individually. First, the inability to access follicles may indeed be true for endometriomas which are larger than 4–5 cm in mean diameter. With regards to exposure to endometrioma fluid, there is no evidence to suggest this is the case. Indeed, at least one investigative team has shown that exposure of oocytes to endometrioma fluid has no impact on rates of fertilisation on early embryo development [6]. Finally, with regards to improving IVF outcome, there are two meta-analyses that have assessed the impact of endometrioma resection on IVF outcomes. Tsoumpou et al. demonstrated no significant differences in response to gonadotropin stimulation or in clinical pregnancy rates, when analyzing five studies which compared surgical resection of endometrioma to no treatment [50]. A Cochrane meta-analysis involving 312 patients by Benschop et al. confirmed that surgical management of endometrioma’s resulted in no benefits for a subsequent IVF cycle [51]. Importantly, these trials are limited as they are surgical in nature, and did not control for any confounding factors with regards to differing surgical techniques (aspiration, stripping and total excision, partial resection, and ablation), endometrioma size, or laterality. Indeed, this may mean that the only indication for removing an endometrioma greater than 3 cm in mean diameter before IVF, as suggested by Elter and Oral, would be to treat painful symptoms or to improve ovarian access [52]. Garcia-Velasco and Somigliana suggested indications for surgical intervention that may be beneficial for assisted reproduction. Proposed Indications for Resection of a Suspected Endometrioma prior to assisted reproduction [13]:

1. rapid growth,
2. suspicious features noted on ultrasound,
3. painful symptoms that can be attributed to the mass,
4. potential for rupture in pregnancy,
5. inability to access follicles in normal ovarian tissue.

Fundamentally, it is crucial that if endometrioma resection is indicated, one must proceed conservatively to minimize any compromise of ovarian blood supply and preserve normal ovarian tissue [53].

Needless to say, there are arguments against pre-cycle treatment of endometrioma. Evidence has suggested that not only has excision of endometriomas failed to be beneficial, but surgery may indeed, be detrimental. The evidence for this statement is based on excision of stable lesions at least 3 cm in diameter and without worrying features [54]. Somigliana et al. reported a 53% reduction in response to gonadotrophins in ovaries that had been operated upon regardless of size of the cyst with an absence in follicular development in 13% of cases after excision of unilateral endometriomas [36, 54]. These data are supported by other studies. Furthermore, Somigliana et al. reviewed that nine of 11 studies showed a statistically significant postoperative decline in serum anti-Mullerian hormone (AMH) levels, which was exacerbated by excision of bilateral lesions [55]. Muzii et al., in a recent meta-analysis, extracted data on 597 patients from 13 evaluated studies, and demonstrated that despite heterogeneity amongst the studies, the antral follicle count was inherently lower in the affected ovary [56].

Harb et al. suggest from their systematic review that the implantation and clinical pregnancy rate are reduced in women with severe endometriosis although the most important clinical outcome was live birth rate, and although a reduction of 14% in live births (RR = 0.86, 95% CI 0.68–1.08) was observed with stage III/IV endometriosis, this did not reach statistical significance [57]. They suggest that this may be attributed to fewer reports of live birth rate in the literature, and hence weakening the power of their review to detect this outcome [57].

Furthermore, Hong et al. reported that IVF cycle outcomes including clinical pregnancy and live birth rate were not significantly different between the two groups of diminished ovarian reserve with surgery and without surgery [58]. They speculate that endometriosis-related infertility is attributed to diminished ovarian reserve and not the reduced endometrial receptivity, inferior oocyte and embryo quality [58].

Contrasting reports have shown that pre-cycle surgical intervention may be beneficial. Opøien et al. studied patients with stage I/II endometriosis from a single centre, in a retrospective trial, who underwent surgical resection or controls who underwent diagnostic laparoscopy only before IVF/ICSI [59]. They found significantly higher clinical pregnancy (40.1 versus 29.4%, *P* = 0.004), implantation (30.9 versus 23.9%; *P* = 0.02) rates were achieved in those who underwent resection than those who underwent diagnostic laparoscopy, and live birth rate per ovum retrieval (27.7 versus 20.6%, *P* = 0.04) [59]. Barri et al. evaluated 825 patients with endometriosis-related infertility over a seven-year period, and reported that overall pregnancy rates were significantly higher in patients undergoing surgical resection and then IVF in comparison to those who underwent surgery alone, IVF alone, or no treatment (65.8, 54.2, 32.1, and 11.8%) [60]. Of note, it was unexpected that pregnancy rates from surgery alone would be so much higher than with IVF alone; however, this may be attributed to pregnancy rates being reported as cumulative. The mean time to achieve pregnancy after surgery was 11.8 ± 12.1 months (range 1–66 months) [60].

The lack of randomised trials regarding pre-IVF cycle surgical management of endometriosis makes it difficult to recommend this approach unless symptom relief is the primary goal.

The current endometriosis guidelines by ESHRE 2013 recommend that “In infertile women with ovarian endometrioma undergoing surgery, clinicians should perform excision of the endometrioma capsule, instead of drainage and electrocoagulation of the endometrioma wall, to increase spontaneous pregnancy rates.” Hart et al. are the source quoted for this statement, but they did not examine within their study, if there is a favoured surgical approach, if any, to women undergoing fertility treatment [61].

These studies highlight that as clinicians, we need to balance the risks and benefits of pre-IVF cycle endometrioma resection given that the bulk of evidence suggests that there is no significant difference in live pregnancy rate. Patients should be counseled with regard to outcome, as well as the risks to ovarian reserve and response particularly in those who already have evidence of compromise.

## Conclusion

Current evidence suggests that women with endometriosis-related infertility have similar cycle outcomes to other patients going through ART. Pre-cycle surgical management of endometrioma does not appear to be beneficial aside from achieving symptom relief, although heterogeneity amongst studies make data analysis challenging.

Endometriomas should not be resected to improve ART outcome and much evidence suggests a detrimental effect of surgery on ovarian reserve and response. The indications for surgical intervention should be limited to suspicious features, rapid growth, progressive symptoms, and an inability to aspirate follicles due to the size of the lesion. Conservative surgical approaches taking great care to avoid compromise of normal ovarian tissue and blood supply are critical. Unfortunately, the evidence is largely based on retrospective data.

It is pertinent for clinicians to assess the risks of surgical intervention on ovarian reserve prior to initiating therapy. The need for additional well-designed prospective randomised controlled trial is vital, as only one RCT had been done before. So we are relying on non-randomised data. In the world of evidence-based medicine, we should aim for the highest standard of evidence; there is a need for multi-centre RCT with live birth rate as the primary outcome to allow clinicians care for these patients.
